# Evaluation of bed vacancy and occupancy times as governance parameters in patients admitted to a public intensive care unit

**DOI:** 10.5935/0103-507X.20200070

**Published:** 2020

**Authors:** Marcos Antonio Alves Júnior, Taciana Silveira Passos, Marcos Antonio Almeida-Santos

**Affiliations:** 1 Department of Medicine, Universidade Tiradentes - Aracaju (SE), Brazil.; 2 Postgraduate Program in Health and Environment, Universidade Tiradentes - Aracaju (SE), Brazil.; 3 Tiradentes Institute, University of Massachusetts, Boston, United States.

**Keywords:** Clinical governance, Health services administration, Bed occupancy, Health status disparities, Intensive care units, Governança clínica, Administração de serviços de saúde, Disparidades em saúde, Ocupação de leitos, Unidades de terapia intensiva

## Abstract

**Objective:**

To evaluate the vacancy and occupancy times of intensive care unit beds; to analyze differences in these times between the day and night shifts and weekdays, weekends, and holidays; and to identify predictors of vacancy and occupancy times.

**Methods:**

This was a cross-sectional, observational, descriptive, analytical, inferential study. A total of 700 vacancy-to-occupancy records from 54 beds of an adult intensive care unit of a public hospital in Sergipe, Brazil, dated between January and December 2018 were analyzed. The nonparametric Mann-Whitney test was used for comparisons between groups. Several predictive models of length of stay were constructed. The incidence rate ratio was used to estimate the effect size.

**Results:**

During the study period, there were 13,477 requests for the 54 intensive care unit beds, and only 5% (700 patients) were granted. The vacancy-to-occupancy times were shorter when beds were occupied at night (incidence rate ratio of 0.658; 95%CI 0.550 - 0.787; p < 0.0001) or on weekends (incidence rate ratio of 0.566; 95%CI 0.382 - 0.838; p = 0.004). Female sex (incidence rate ratio of 0.749; 95%CI 0.657 - 0.856; p < 0.0001) was a predictor of shorter vacancy-to-occupancy time. This time tended to increase with patient age (incidence rate ratio of 1.006; 95% CI 1.003 - 1.009; p < 0.0001).

**Conclusion:**

Disparities in the waiting time for intensive care unit beds were identified, as the time was greater in the daytime and on weekdays, and women and younger patients experienced shorter vacancy-to-occupancy times.

## Abstract (pt)

**Objetivo:**

Avaliar o tempo de desocupação e ocupação dos leitos na unidade de terapia intensiva; analisar os intervalos entre os tempos durante o período do dia e da noite, finais de semana e feriados e identificar preditores para os tempos de desocupação e ocupação.

**Métodos:**

Estudo transversal, de natureza observacional, descritivo, analítico e inferencial. Foram analisados 700 registros de desocupação-ocupação em 54 leitos na unidade de terapia intensiva adulto de um hospital da rede pública de Sergipe, entre janeiro e dezembro de 2018. O teste não paramétrico de Mann-Whitney foi utilizado para comparações entre grupos. Diversos modelos preditivos de tempo de permanência foram elaborados. A razão de taxa de incidência foi utilizada como estimativa de tamanho do efeito.

**Resultados:**

Durante o período do estudo, houve 13.477 solicitações de vaga na unidade de terapia intensiva para os 54 leitos, e apenas 5% (700 pacientes) conseguiram o acesso ao leito. Os tempos de desocupação-ocupação tiveram valores menores quando a ocupação do leito era realizada no período noturno (razão de taxa de incidência de 0,658; IC95% 0,550 - 0,787; p < 0,0001) e oferta nos finais de semana (razão de taxa de incidência de 0,566; IC95% 0,382 - 0,838; p = 0,004). O sexo feminino (razão de taxa de incidência de 0,749; IC95% 0,657 - 0,856; p < 0,0001) foi um preditor de menor tempo de desocupação-ocupação. Esse tempo tende a aumentar com a idade do paciente (razão de taxa de incidência de 1,006; IC95% 1,003 - 1,009; p < 0,0001).

**Conclusão:**

Identificaram-se disparidades no tempo de espera para a ocupação do leito, sendo maior no período diurno e em dias úteis. Mulheres e pacientes mais jovens são beneficiados por um processamento mais rápido no tempo de desocupação-ocupação.

## INTRODUCTION

Clinical governance is an organized system whose commitment is to provide high-quality care for clinical practice. It is responsible for the continuous improvement of service quality and for keeping quality parameters high. In the past, administrative meetings were mainly concerned with financial issues and activity targets. With the advent of evidence-based medicine, the concept of successful administration was expanded to fully integrate financial control, service performance, and high clinical quality.^(1)^

Intensive care units (ICUs) are places of high investment within the hospital, whose resources should be allocated as best as possible to meet the needs of critically ill patients. To this end, an administrative unit should be formed that is knowledgeable of hospital resources and that determines which objective criteria should be adopted for both ICU admission and discharge, enabling the control of patient flow and the permanent monitoring of compliance with established protocols.^(2)^ For this purpose, the Internal Regulation Committee (*Núcleo Interno de Regulação* - NIR), a technical-administrative unit of the Unified Health System (*Sistema Único de Saúde* - SUS), was created. The NIR is responsible for monitoring patients from hospital admission through their internal and external movements to hospital discharge.^(3)^

Studies on clinical governance are potentially relevant to health service managers, both in the public and private spheres, as they can evaluate the performance and quality of the service as a management tool. In regard to the ICU, the proper triage of patients is a critical requirement for the proper use of hospital resources, and it is even more important in an environment with limited beds. Because ICUs are considered high complexity, that they need proportionally more human and material resources than other hospital sectors.^(4-6)^

Considering the extent to which it can be investigated in national and international databases, few studies have identified the factors affecting the length of the entire time between bed vacancy and effective reoccupancy. The objective of this study was to evaluate the vacancy-to-occupancy time of beds in an adult ICU.

## METHODS

This was a cross-sectional, observational, descriptive, analytical, and inferential study. The sample consisted of the 54 beds of an adult ICU in a large hospital in the state of Sergipe, Brazil, whose admission records were tracked by the NIR. The adult ICU is spatially divided into two floors, each with 27 beds. Thus, it was agreed to call the ICU on the ground floor ICU 1 and the ICU on the upper floor ICU 2, but there was no difference in the clinical-epidemiological profile of patients admitted to them. The medical staff consisted of four hospitalists and four nurses per shift for each adult ICU space. The unit had six attending physicians.

A total of 700 vacancy-to-occupancy records corresponding to the adult ICU beds dating from 1 January to 31 December 2018 were analyzed. The computer records of the vacancy-to-occupancy processes of beds in the adult ICU during the study period were included. Computer records with incomplete, inappropriate, and/or conflicting data were excluded.

The research data were obtained from the NIR, which operates 24 hours a day, 7 days a week. It tracks in real time the available adult ICU beds and the list of patients waiting for a bed. This information is sent to the State Regulatory Complex, a component of the Interstate Universal Access Guarantee System (*Sistema Interfederativo de Garantia de Acesso Universal* - SIGAU), which authorizes the occupancy of the bed.

The criteria for admission to the adult ICU were age ≥13 years, proper completion of the Bed Request Application, and receipt of an authorization code from the Bed Regulation Center (*Central de Regulação de Leitos* - CRL). Patients are categorized by the NIR physician into degrees of priority for access to ICU beds according to the Federal Council of Medicine (*Conselho Federal de Medicina* - CFM) resolution no. 2,156/2016.^(7)^

The data collected were entered into an Excel spreadsheet, in which each shift was categorized as night, weekend, and/or holiday and the vacancy-to-offer, offer-to-occupancy, and vacancy-to-occupancy times were calculated for the different shifts. Time is expressed in minutes to standardize the measurements.

The vacancy-to-offer time corresponded to time between when the bed was vacated to when it was offered to another patient. The offer-to-occupancy time was the time between when the bed was offered to a patient and the time when the patient occupied the bed. The vacancy-to-occupancy time referred to the total time between when the bed was vacated and effectively occupied again, being the sum of the other two times.

Based on the working hours of the shifts, the following shifts were defined for the purposes of present study: night shift, 7:00 p.m. to 6:59 a.m.; weekend shift, 7:00 p.m. Friday to 6:59 a.m. Monday; and holiday shift, 7:00 p.m. on the day before the holiday to 6:59 a.m. on the first day after the holiday. A total of 23 public holidays and/or optional days off that were relevant the study hospital were recorded.

### Data analysis

Categorical variables are expressed as absolute numbers and percentages. Discrete numerical variables are expressed as the median and interquartile range (IQR). The nonparametric Mann-Whitney test was used for comparisons between groups.

Several predictive models of length of stay were constructed, including Poisson and negative binomial regressions. In situations in which the length of stay did not assume null values, the estimates were corrected for zero-truncation. Type II negative binomial regression was considered the best-fitting model. This predictive model allows adjustment for overdispersion, a phenomenon often found in time measurements; presented a significant value for the alpha parameter in the likelihood ratio test, suggesting that the Poisson regression needed to be disregarded in these cases; produced a p-value > 0.05 in Pregibon’s link test, indicating adequate specification; and presented global and comparative parameters of satisfactory model fit, such as lower Akaike’s (AIC) and Bayes’s information criterion (BIC) values.

The incidence rate ratio (IRR) was used to estimate the effect size. The IRR is used to evaluate the effect of one category in relation to another, similar to the interpretation of the odds ratio, but for count data. The vacancy-to-occupancy time was the dependent variable.

To evaluate the dispersion of the measurements and because it shows greater efficiency and robustness when the asymptotic distribution assumption is not valid (as in the case of small samples), the observed information matrix was used to calculate the standard error and 95% confidence intervals (95%CI). A p value < 0.05 was adopted as the statistical significance level. The calculations were performed in the Stata statistical program (College Station, Texas, USA), version 15.1.

The study followed the guidelines and regulatory standards of research set by the Ministry of Health’s National Health Council resolution no. 466 of December 12, 2012 and was approved by the Research Ethics Committee (REC) of *Universidade Tiradentes* under CAAE no. 90468718.8.0000.5371. The free and informed consent form was waived by the REC, as this was a retrospective survey of data in medical records.

## RESULTS

**Figure 1:**
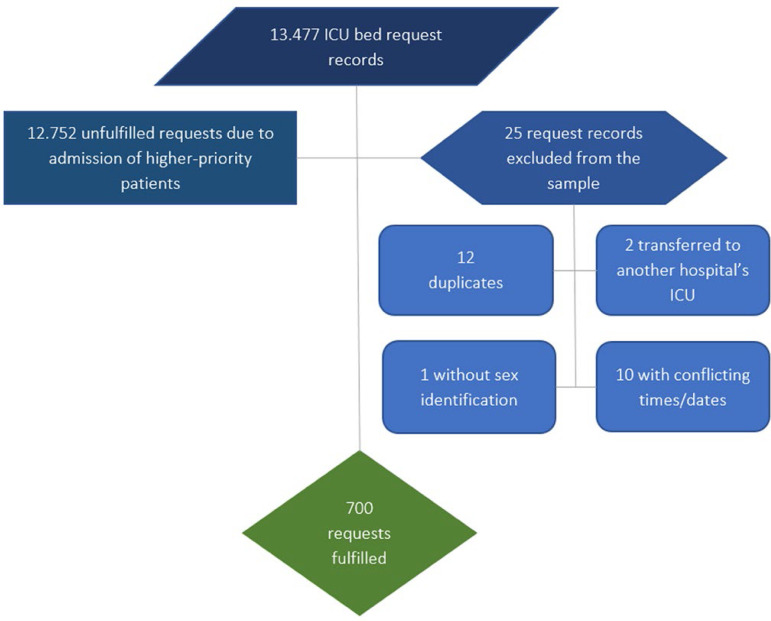
Flowchart of sample selection. ICU - intensive care unit.

During the study period, there were 13,477 requests for the 54 ICU beds. Only 700 requests were granted, corresponding to 5%. Note that the number of requests did not equal the number of patients. The requests were made daily, and if a patient had not yet been transferred, a new request was made the next day. The number of patients who could not be transferred to the ICU was not available. All records whose vacancy and/or occupancy data were incomplete, inappropriate, and/or conflicting were excluded from the study (Figure 1).

Access to the ICU beds was regulated within the hospital by nine physicians working in the NIR, and in their absence, the SIGAU assumed responsibility for admission. Most patients (98%) were from internal areas of the hospital, mainly from the Post-Anesthesia Recovery Room (43%) and from the Red Area (emergency ward) (40%).

There was no difference in the percentage of bed occupancy between the ICUs, with ICU 1 responsible for 322 (46%) admissions and ICU 2 responsible for 378 (54%). The study population consisted of 434 (62%) men and had a median age of 50 years (IQR 33 - 65). Almost all patients admitted to the adult ICU during the study period had the same priority clinical profile (Table 1).

**Table 1:** Profile of patients admitted to a public intensive care unit

Variables	Value
Sex	
Female	266 (38)
Male	434 (62)
Age, median (IQR)	50 (33 - 65)
ICU	
ICU 1	322 (46)
ICU 2	378 (54)
Clinical profile of hospitalized patients	
Priority 1	665 (98.52)
Priority 2	8 (1.19)
Priority 3	1 (0.15)
Priority 4	1 (0.15)

IQR - interquartile range; ICU - intensive care unit. Results expressed as n (%).

When comparing the work shifts, the daytime during a weekday (7 a.m. to 7 p.m.) was considered the reference time. The times elapsed during other shifts (nights, weekends, and holidays) were compared to this reference time. Among the binomial variables, the vacancy-to-occupancy time was significantly shorter when the occupancy occurred during the night shift (median of 407; IQR 307 - 615; p < 0.0001). The vacancy-to-offer and offer-to-occupancy times were also significantly shorter (p < 0.0001) when the bed was offered at night. When the bed was offered on the weekend, the vacancy-to-occupancy time was shorter (median of 472.5; IQR 325 - 770; p = 0.028) than during the week. However, the vacancy-to-occupancy time was significantly longer when the bed was offered (median of 700; IQR 400 - 1475; p = 0.024) or occupied on a holiday (median of 886; IQR 435 - 1560; p = 0.001) (Table 2).

**Table 2:** Vacancy-to-offer, offer-to-occupancy, and vacancy-to-occupancy times according to time of day, day of week, and holiday or not in a public intensive care unit

	Times/shift*	Night		p value	Weekend		p value	Holiday		p value
		Median (IQR)			Median (IQR)			Median (IQR)		
		No†	Yes		No	Yes		No	Yes	
Shift of offer	Vacancy-to-offer time	580 (340 - 990)	309 (215 - 430)	< 0.0001	383 (255 - 811)	347 (230 - 617.5)	0.024	370 (245 - 705)	434 (345 - 1055)	0.033
	Offer-to-occupancy time	90 (50 - 165)	80 (45 - 150)	0.066	80 (45 - 153)	85 (46 - 170)	0.358	82 (45 - 155)	92 (52 - 260)	0.162
	Vacancy-to-occupancy time	755 (450 - 1170)	407 (307 - 615)	< 0.0001	525 (350 - 1030)	482 (335 - 765)	0.051	495 (336 - 900)	886 (425 - 1560)	0.001
Shif of occupanc	Vacancy-to-offer time	501 (303 - 963)	318 (217 - 420)	< 0.0001	385 (255 - 805)	347 (230 - 625)	0.034	370 (245 - 710)	425 (300 - 990)	0.118
	Offer-to-occupancy time	115 (53 - 194)	65 (40 - 105)	< 0.0001	84 (46 - 161)	84 (45 - 150)	0.580	83 (45 - 155)	90 (300 - 990)	0.565
	Vacancy-to-occupancy time	690 (440 - 1120)	385 (300 - 560)	< 0.0001	52 4(350 - 1000)	47 3(325 - 770)	0.028	490 (336 - 880)	700 (400 - 1475)	0.024

IQR - interquartile range.

* Mann-Whitney test;

† time elapsed on a weekday during the day.

The Poisson regression models showed high overdispersion values. As a result, negative binomial regression models were adopted, which showed alpha values > 0.8 and a significant outcome of the likelihood ratio test (p < 0.0001), in addition to lower AIC and BIC values. Postestimations of the three models indicated satisfactory specification according to Pregibon’s link test, with nonsignificant p-values when quadratic terms were added as predictors.

In the analysis of the predictive model, being female decreased the vacancy-to-occupancy time significantly (IRR of 0.749; 95% CI 0.657 - 0.856; p < 0.0001) (Table 3). Other models, constructed for the vacancy-to-offer and offer-to-occupancy times, showed consistency in the lower times for women.

**Table 3:** Predictive model for the vacancy-occupancy time in an intensive care unit of the public health network

	IRR	95%CI	p value
Sex			
Male (reference)			
Female	0.749	0.657 - 0.856	< 0.0001
Age	1.006	1.003 - 1.009	< 0.0001
ICU			
ICU 1 (reference)			
ICU 2	0.944	0.830 - 1.073	0.382
Occupancy on weekends	1.441	0.982 - 2.113	0.061
Occupancy on holidays	1.198	0.776 - 1.850	0.413
Occupancy on nights	0.658	0.550 - 0.787	< 0.0001
Offer on weekends	0.566	0.382 - 0.838	0.004
Offer on holidays	1.268	0.836 - 1.923	0.263
Offer on nights	0.834	0.696 - 1.000	0.050

IRR - incidence rate ratio; 95% CI - 95% confidence interval; ICU - intensive care unit.

For each additional year of patient age, the vacancy-to-occupancy time increased significantly (IRR of 1.006; 95%CI 1.003 - 1.009; p < 0.0001). The vacancy-to-occupancy time was not affected by which ICU the patient was referred to. However, beds were occupied significantly faster at night (IRR of 0.658; p < 0.0001; 95%CI 0.550 - 0.787).

**Figure 2:**
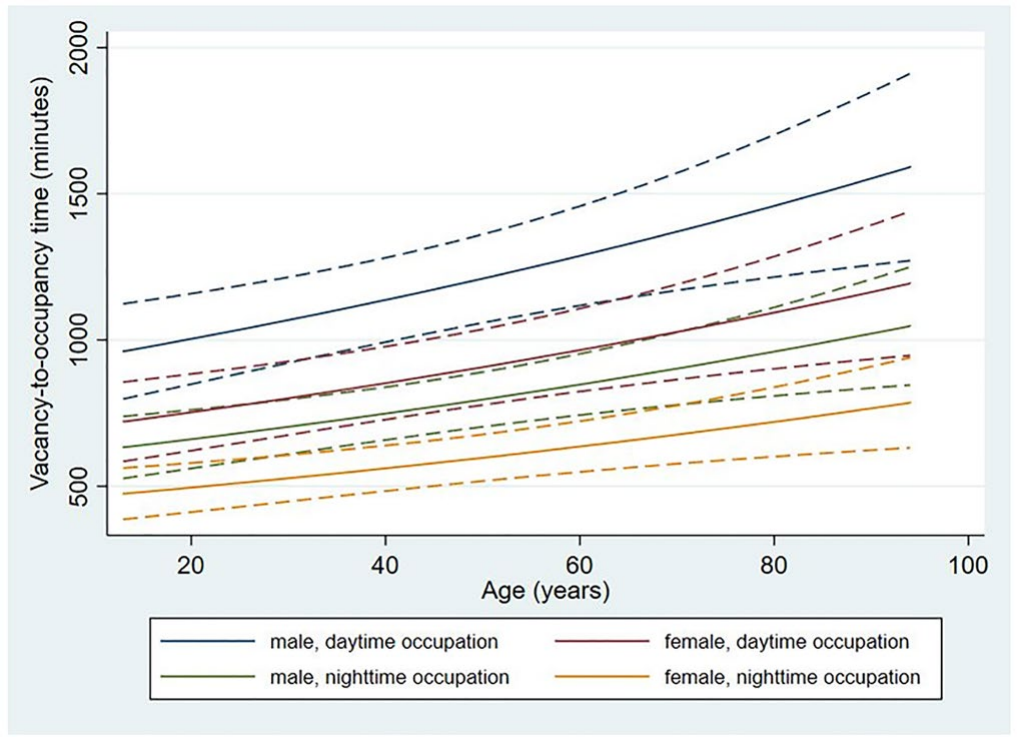
Predictive values with confidence intervals for vacancy-to-occupancy time according to age, sex, and shift of bed occupation.

The vacancy-to-occupancy time was longer when the occupancy occurred during the day, regardless of patient sex. However, men experienced even longer times than women for the same shift of occupancy. The vacancy-to-occupancy time increased with the age of the admitted patient (Figure 2).

## DISCUSSION

This study shows that there was an overload of requests for ICU beds. Among the different times and days of the week, shorter vacancy-to-occupancy times of beds were found at night. Occupancy was also faster at night (p < 0.0001). This finding differs from other studies, in which longer times for processes performed at night and on weekends were observed, explained by fewer staff in those periods.^(8-11)^ However, the of vacancy-to-occupancy time increased when the bed was offered on holidays, in line with the cited studies.

The vacancy-to-occupancy processing of the ICU beds occurred more quickly at night due to the switch to the night-shift team. This team may be more quickly organizing patients whose access to the ICU has already been authorized, reducing the number of pending issues to be resolved during its shift and thus providing faster care to patients. Another factor that may be implied is that there are more activities to be performed by staff in the daytime, which keeps them occupied with various other tasks.

Regarding the differences by patient sex, fewer females (38%) were admitted to the adult ICU than males, a result similar to that found in other studies.^(6,12,13)^ The criteria for ICU admission were not influenced by sex but rather by objective parameters, and the population of the present study had a homogeneous priority profile. Nevertheless, there was a sex disparity in the times measured in this study, since women had significantly shorter (p < 0.0001) vacancy-to-occupancy times. This finding may have resulted from a subjective evaluation by the staff, who may provide better and more attentive care when the patient is female than male, considering that there is no internal service policy that can explain this difference. Other studies have also shown that being female is a protective factor for ICU admission, resulting in better outcomes. This factor, however, could be associated with hormonal differences between the sexes and not with the speed of care.^(13,14)^

Regarding age, for each additional year of life, the vacancy-to-occupancy time increased significantly (IRR of 1.006; 95%CI 1.003 - 1.009; p < 0.0001). Thus, younger patients had a shorter ICU bed occupancy processing time. Although age was not one of the priority criteria used, older patients do have lower functional reserve. This factor may result, unconsciously, in faster processing times for young patients because staff may believe that they will have a better outcome, ignoring the similarity in clinical profile.

The limiting factors of the study were the failure to evaluate the relationship between the time to access the adult ICU bed or the clinical outcomes of patients and the fact that patients admitted after surgery and those with purely clinical needs were analyzed together. Even so, we believe that the times would have been similar if these groups had been analyzed separately. The response time of the SIGAU to release the code authorizing the occupancy of the bed was also not evaluated, so the vacancy and occupancy times may also be subject to factors external to the hospital. Reliability in filling the patient’s priority level is another limiting factor, since when seeking faster care, the physician may allocate the patient to a category other than the one dictated by the patient’s presentation.

The strengths of this study are the reporting of objective data that show a difference in health care provision by the work shift and day of the week in which the process occurred and by patient sex and age. Thus, managers should determine which parameters will let them better adjust the care provided, particularly because the ICU is a highly complex service with a high usage of human and financial resources.

## CONCLUSION

The study identified disparities in the waiting times for beds in the adult intensive care unit. The waiting time was longer during the daytime, and women experienced shorter vacancy-to-occupancy times. Another protective factor for a faster admission was younger patient age.

Phenomena related to differences in the number of staff and in their proactivity according to the work shift may have influenced the measurements. Future studies should determine the reasons why women and younger patients occupied the adult intensive care unit beds more quickly despite presenting the same clinical parameters as men and older patients.
